# Quantification of C4d deposition and hepatitis C virus RNA in tissue in cases of graft rejection and hepatitis C recurrence after liver transplantation

**DOI:** 10.1590/0074-02760140192

**Published:** 2015-02

**Authors:** Alice Tung Wan Song, Evandro Sobroza de Mello, Venâncio Avancini Ferreira Alves, Norma de Paula Cavalheiro, Carlos Eduardo Melo, Patricia Rodrigues Bonazzi, Fatima Mitiko Tengan, Maristela Pinheiro Freire, Antonio Alci Barone, Luiz Augusto Carneiro D'Albuquerque, Edson Abdala

**Affiliations:** 1 Divisão de Transplante de Fígado e Órgãos do Aparelho Digestivo; 2 Laboratório de Transplante de Fígado - LIM 37; 3 Departamento de Patologia; 4 Laboratório de Hepatites Virais - LIM 47; 5 Divisão de Clínica de Moléstias Infecciosas e Parasitárias; 6 Subcomissão de Infecção Hospitalar, Hospital das Clínicas, Universidade de São Paulo, SP, Brasil

**Keywords:** complement, humoral, PCR, tissue, immunohistochemistry

## Abstract

Histology is the gold standard for diagnosing acute rejection and hepatitis C recurrence after liver transplantation. However, differential diagnosis between the two can be difficult. We evaluated the role of C4d staining and quantification of hepatitis C virus (HCV) RNA levels in liver tissue. This was a retrospective study of 98 liver biopsy samples divided into four groups by histological diagnosis: acute rejection in patients undergoing liver transplant for hepatitis C (RejHCV+), HCV recurrence in patients undergoing liver transplant for hepatitis C (HCVTx+), acute rejection in patients undergoing liver transplant for reasons other than hepatitis C and chronic hepatitis C not transplanted (HCVTx-). All samples were submitted for immunohistochemical staining for C4d and HCV RNA quantification. Immunoexpression of C4d was observed in the portal vessels and was highest in the HCVTx- group. There was no difference in C4d expression between the RejHCV+ and HCVTx+ groups. However, tissue HCV RNA levels were higher in the HCVTx+ group samples than in the RejHCV+ group samples. Additionally, there was a significant correlation between tissue and serum levels of HCV RNA. The quantification of HCV RNA in liver tissue might prove to be an efficient diagnostic test for the recurrence of HCV infection.

Advanced liver disease caused by hepatitis C virus (HCV) infection is the leading indication for liver transplantation worldwide (Saab & Wang 2003, Roche & Samuel 2007). The post-transplant detection of HCV RNA in the serum or graft is extremely common, occurring in more than 95% of cases (Berenguer 2002, Roche & Samuel 2007). In more than half of those cases, infection recurs within the first year after transplantation. In transplant recipients, the disease is particularly aggressive, with rapid progression of fibrosis (Berenguer 2002). During post-transplant follow up, elevated liver enzymes warrant liver biopsies for accurate diagnosis and treatment and it can be difficult to differentiate between acute rejection and recurrence of hepatitis C. Although histological evaluation is the gold-standard method for distinguishing between the two entities, there have been reports of misdiagnosis because of overlapping morphological features (McCaughan & Zekry 2002, Leung et al. 2003, Regev et al. 2004).

Following activation of the complement system, the C4d fragment forms a covalent bond with tissues and C4d immunostaining has been widely used to demonstrate antibody-mediated rejection of organ transplants (Michaels et al. 2003). There is evidence that humoral mechanisms are involved in the pathogenesis of acute rejection in liver transplant recipients (Takakura et al. 1999, Krukemeyer et al. 2004, Sawada et al. 2005, Bu et al. 2006 ). Recent studies have correlated concurrent donor-specific human leukocyte antigen (HLA) antibody detection with the histological features of this form of rejection and C4d immunostaining (Bellamy et al. 2007 , Sakashita et al. 2007, Aguilera et al. 2011, Kozlowski et al. 2011 , 2012, Musat et al. 2011, Lunz et al. 2012). In some studies in liver transplantation, C4d staining has been shown to be useful as a complementary method for discriminating between graft rejection and the recurrence of hepatitis C (Jain et al. 2006, Lorho et al. 2006, Schmeding et al. 2006 , 2010). Studies of correlations between serum HCV RNA levels and the recurrence of hepatitis C have indicated that the determination of serum HCV RNA can also be used to discriminate between these two diagnoses (Fragulidis et al. 1998, Aardema et al. 1999, Gottschlich et al. 2001, D'Errico-Grigioni et al. 2008).

In the present study, we aimed to evaluate C4d immunostaining and quantification of HCV RNA in tissue, including their utility in differentiating hepatitis C recurrence from acute rejection in cases of acute rejection in patients with and without HCV infection, HCV recurrence and chronic hepatitis C in the non-transplant setting. We also attempted to determine whether C4d deposition correlated with epidemiological, clinical and histological features of acute rejection and hepatitis C recurrence, as well as whether the level of HCV RNA in tissue correlated with the histological features of chronic hepatitis.

## PATIENTS, MATERIALS AND METHODS

*Study design* - This study was performed using liver biopsies from patients who had undergone liver transplantation or from outpatients with chronic hepatitis C. All of the biopsies evaluated had been performed between 1998-2011 at the University of São Paulo School of Medicine (FMUSP) Clinics Hospital. A local ethical committee approved the study.

*Patients* - The specimens were initially obtained from a list of histological diagnoses and reviewed by two pathologists with expertise in the field who were blinded to the clinical diagnoses. We selected biopsy samples that met the following eligibility criteria: obtained from biopsies performed within the first year after the transplant (when applicable); had only one sample from each patient and contained six or more portal tracts, with four or more centrilobular hepatic veins. We excluded biopsy samples obtained from patients with hepatitis B, autoimmune hepatitis, primary biliary cirrhosis, primary sclerosing cholangitis or storage diseases for all groups. We also excluded samples in which signs of rejection and hepatitis C recurrence were both found, as they were not related to this study's objective.

On the basis of the histological diagnosis, we divided the biopsy samples into four groups: acute rejection in recipients of liver transplants performed because of HCV-related cirrhosis (RejHCV+), recurrence of hepatitis C in recipients of liver transplants performed because of HCV-related cirrhosis (HCVTx+), acute rejection in recipients of liver transplants performed for reasons other than HCV infection (RejHCV-) and chronic hepatitis C patients in a non-transplant setting (HCVTx-).

On the basis of those criteria, we selected 98 formalin-fixed, paraffin-embedded liver tissue samples for inclusion in the study.

Recurrence of hepatitis C was defined as the post-transplant presence of HCV RNA in serum and chronic portal inflammation, with or without portal lymphoid aggregates, together with necroinflammatory and ductular-type interface activity of varying severity (Demetris 2009). For the grading and staging of chronic hepatitis, the modified Ishak classification was used (Ishak et al. 1995). Acute rejection was defined as inflammation of the graft, primarily affecting the interlobular bile ducts and vascular endothelia, including the portal veins and hepatic venules, with or without involvement of the hepatic artery and its branches (Banff schema for grading liver allograft rejection: an international consensus document 1997). For the grading and staging of acute rejection, the Banff criteria were used (Banff schema for grading liver allograft rejection: an international consensus document 1997).

*Clinical and laboratory data* - Patient charts were reviewed and the following data were collected (when applicable): age, gender, time from transplantation to biopsy (arbitrarily divided into intervals), living or deceased donor, donor age, main diagnosis before transplantation, pre-transplant or pre-biopsy use of interferon (IFN), total ischemia time, pre-transplant or pre-biopsy serum HCV RNA, immunosuppressive drugs and HCV genotype.

*Immunohistochemical staining for C4d* - All of the specimens were subjected to immunohistochemical staining for C4d and the same two pathologists performed the quantitative grading for all the specimens. All of the fields were analysed and positivity was defined as clear-cut immunostaining of endothelial cell membranes of each vascular component, specifically portal veins, sinusoids and (centrilobular) hepatic veins. Due to the current debate about the specificity of C4d immunoreactivity of the hepatic artery, C4d staining in the hepatic artery was not considered indicative of positivity, nor was stromal staining for C4d.

In brief, 3-µm tissue sections were deparaffinised, unmasked and stained with a commercially available polyclonal antibody against C4d (BI-RC4D, 1:50; Biomedica, Austria). Heat-induced epitope retrieval was optimised with the EDTA/TRIS buffer, pH 8.0, for 40 min in a steamer. Amplification was performed using the polymer-peroxidase complex (Novolink Max Polymer; Novocastra Laboratories, UK). As the chromogen for the peroxidase reaction, we used 3,3'-diaminobenzidine (Dako, Denmark). Counter-staining was performed with Harris haematoxylin and the endogenous peroxidase was blocked using hydrogen peroxide. The positive controls consisted of kidney biopsy samples with known antibody-mediated rejection and the negative controls consisted of samples stained without the primary antibody.

*HCV RNA quantification* - Specimens from the RejHCV+, HCVTx+ and HCVTx- groups were subjected to HCV RNA quantification by polymerase chain reaction (PCR). The HCV RNA extraction was performed on 10-µm samples of formalin-fixed, paraffin-embedded tissue sections using a commercially available kit (High Pure RNA Paraffin Kit; Roche Diagnostics GmbH, Germany), as per the standardised protocol. Microtubes containing the eluted RNA were stored at -80ºC until RNA amplification.

For real-time amplification, we used a commercial kit (COBAS^(r)^ TaqMan^(r)^ HCV; Roche Diagnostics GmbH) according to the standardised protocol. The reaction was performed in a Cobas Taqman 48 analyser (Roche Molecular Systems, USA) and was analysed using the Amplilink software v.3.2 (Roche Molecular Systems). The lower and upper limits of detection were 25 IU mL^-1^ and 3.91 × 10^8^ IU mL^-1^, respectively.

Negative and positive controls consisted of specimens from patients who had undergone transplantation for primary biliary cirrhosis and of known HCV RNA-positive samples, respectively. All reactions were performed only once.

*Statistical analysis* - The sample size was calculated on the basis of the prevalence of C4d positivity, as reported in a previous study (Schmeding et al. 2006), which was 67% for acute rejection and 12% for hepatitis C recurrence. Using two-sample tests for proportion comparisons with a significance level of 0.05 and a power of 0.8, we obtained prevalence values of 0.55, 0.6, 0.65, 0.7 and 0.75 for acute rejection and of 0.05, 0.1, 0.15 and 0.2 for hepatitis C recurrence; all possible combinations were compared. It was determined that there should be at least 28 cases in each of the study groups (RejHCV+ and HCVTx+) and at least 22 cases in each of the control groups (RejHCV- and HCVTx-).

Quantitative variables were described using measures of central tendency and dispersion and were compared using the Kruskal-Wallis test, followed by Dunn's multiple comparison test or analysis of variance, followed by Tukey's multiple comparison test. Qualitative variables were described using absolute and relative frequencies and compared using chi-square tests or likelihood ratios. For ordinal qualitative variables, we used the Mann-Whitney *U* or Kruskal-Wallis test. Variables showing statistical significance in the univariate analysis were included in logistic regressions for multivariate analysis. Spearman's correlation coefficient was calculated for both diagnostic tests with qualitative and ordinal variables. For variables showing statistical significance, a linear regression model was created for multivariate analysis. For the PCR analysis of HCV RNA, we constructed a receiver operating characteristic (ROC) curve. The statistical analysis was performed using the Statistical Package for the Social Sciences v.15.0 for Windows (SPSS Inc, USA) and the level of significance was set at p < 0.05 for all tests.

*Ethics* - The procedures followed were in accordance with the ethical standards of the responsible institutional committee on human experimentation and with the Helsinki Declaration of 1975, as revised in 1983.

## RESULTS

*Demographic, clinical and laboratory data* - Ninety-eight biopsy samples were selected: 28 cases in the RejHCV+ group, 25 cases in the HCVTx+ group, 20 cases in the RejHCV- group and 25 cases in the HCVTx- group. The baseline characteristics of each group are shown in Tables I, II. There were significant differences among the groups in terms of the mean patient age at biopsy (p = 0.005): in the multiple comparison test, a statistically significant difference was found between the HCVTx+ and RejHCV- groups (54 years vs. 44.5 years, p = 0.02), as well as between the HCVTx+ and HCVTx- groups (54 years vs. 45.5 years, p = 0.03).

**TABLE I:** Qualitative baseline characteristics of all casesa

		Group n (%)					
							
Variable	Category	RejHCV+	HCVTx+	RejHCV-	HCVTx-	Total	p
Gender	Male	17 (60.7)	18 (72)	9 (45)	12 (48)	56 (57.1)	NS^*b*^
	Female	11 (39.3)	7 (28)	11 (55)	13 (52)	42 (42.9)	
Donor type	Deceased	22 (91.7)	22 (88)	20 (100)	-	64 (92.8)	NS^*c*^
	Living	0 (0)	2 (8)	0 (0)	-	2 (2.9)	
	Domino	2 (8.3)	1 (4)	0 (0)	-	3 (4.3)	
Previous IFN use	No	6 (27.3)	10 (40)	-	19 (82.6)	35 (50)	< 0.001^*b*^
	Yes	16 (72.7)	15 (60)	-	4 (17.4)	35 (50)	
HCV genotype	1	13 (59.1)	17 (68)	-	14 (66.7)	44 (64.7)	NS^*c*^
	2	1 (4.5)	0 (0)	-	0 (0)	1 (1.5)	
	3	8 (36.4)	8 (32)	-	7 (33.3)	23 (33.8)	
Use of MMF or MPA	No	20 (83.3)	18 (72)	17 (85)	-	55 (79.7)	NS^*c*^
	Yes	4 (16.7)	7 (28)	3 (15)	-	14 (20.3)	
Year of biopsy	1998-2006	15 (53.6)	7 (28)	8 (40)	19 (76)	49 (50)	0.006^*b*^
	2007-2011	13 (46.4)	18 (72)	12 (60)	6 (24)	49 (50)	

a: numbers discrepancies are due to missing data (patient charts unavailable); b: chi-square test; c: likelihood ratio test; HCV: hepatitis C virus; HCVTx-: chronic hepatitis C in patients in a non-transplant setting; HCVTx+: hepatitis C recurrence in patients undergoing liver transplant for hepatitis C; IFN: interferon; MMF: mycophenolate mofetil; MPA: mycophenolic acid; NS: non-significant; RejHCV-: acute rejection in patients undergoing liver transplant for reasons other than hepatitis C; RejHCV+: acute rejection in patients undergoing liver transplant for hepatitis C.

**TABLE II:** Quantitative baseline characteristics of the cases by group

Variable	Group	n	Mean (range)	p
Age (years)	RejHCV+	28	52.04 (32-67)	0.005^*a*^
	HCVTx+	25	54.00 (25-69)	
	RejHCV-	20	44.45 (21-68)	
	HCVTx-	25	45.52 (22-62)	
Time from transplantation to biopsy (days)	RejHCV+	28	18.29 (4-95)	< 0.001^*b*^
	HCVTx+	25	205.52 (41-374)	
	RejHCV-	20	41.35 (4-276)	
Donor age (years)	RejHCV+	23	44.96 (22-69)	0.021^*a*^
	HCVTx+	23	51.78 (16-73)	
	RejHCV-	20	40.20 (19-59)	
Total ischemia time (min)	RejHCV+	24	507.25 (186-822)	0.572^*b*^
	HCVTx+	25	458.20 (133-640)	
	RejHCV-	20	483.95 (297-753)	

a: ANOVA; b: Kruskal-Wallis test; HCVTx-: chronic hepatitis C in patients in a non-transplant setting; HCVTx+: hepatitis C recurrence in patients undergoing liver transplant for hepatitis C; RejHCV-: acute rejection in patients undergoing liver transplant for reasons other than hepatitis C; RejHCV+: acute rejection in patients undergoing liver transplant for hepatitis C.

The mean time from transplantation to biopsy was longer in the HCVTx+ group (205 days) than in the RejHCV+ and RejHCV- groups (18 days and 45 days, respectively, p < 0.001 for both). Donor age differed only between the HCVTx+ and RejHCV- groups (p = 0.02). When used, immunosuppression therapy consisted of the administration of tacrolimus and prednisone, with or without mycophenolate mofetil; no differences in immunosuppression were observed between the groups. IFN use was more common in the RejHCV+ and HCVTx+ groups than in the HCVTx- group (p < 0.001 and p = 0.004, respectively).

According to the Banff scores, acute rejection in the RejHCV+ and RejHCV- groups, collectively, was mild in 15 cases (31.3%), moderate in 23 (47.9%) and severe in 10 (20.8%), with no differences between the two groups.

The Ishak staging results for the patients with chronic hepatitis demonstrated that there were more cases of advanced-stage fibrosis, portal inflammation and periportal inflammation in the HCVTx- group than in the HCVTx+ group (p < 0.001, p = 0.004 and p = 0.04, respectively). Parenchymal inflammation was comparable between the two groups (p = 0.37 for confluent necrosis and p = 0.64 for focal lytic necrosis).

*C4d immunostaining* - As seen in Table III, C4d deposition was observed more often in the portal compartment (68.4%) than in the sinusoidal and centrilobular compartments (8.2% and 10.2%, respectively). The C4d deposition observed in the study samples is shown in Figure.

**TABLE III:** Proportion of C4d immunostaining positivity in all groups by hepatic compartment

Group	Portal n/N (%)	Sinusoidal n/N (%)	Centrilobular n/N (%)
RejHCV+	15/28 (53.5)	3/28 (10.7)	3/28 (10.7)
HCVTx+	13/25 (52)	0/25 (0)	0/25 (0)
RejHCV-	12/20 (60)	1/20 (5)	5/20 (25)
HCVTx-	23/25 (92)	4/25 (1.6)	2/25 (8)
p	0.009	NS	NS

HCVTx-: chronic hepatitis C in patients in a non-transplant setting; HCVTx+: hepatitis C recurrence in patients undergoing liver transplant for hepatitis C; NS: non-significant; RejHCV-: acute rejection in patients undergoing liver transplant for reasons other than hepatitis C; RejHCV+: acute rejection in patients undergoing liver transplant for hepatitis C.

**Figure:**
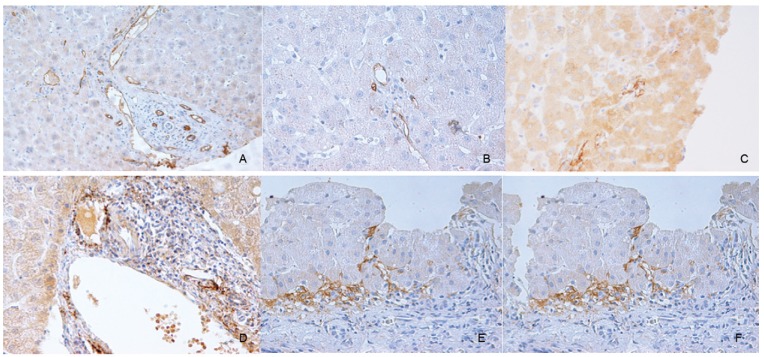
C4d immunostaining. A: acute rejection in patients undergoing liver transplant for hepatitis C (RejHCV+) group case with mild acute rejection (Banff 1/1/1) and strong C4d immunoreactivity in the endothelial cells of portal veins (400X); B: RejHCV+ group case with moderate acute rejection (Banff 2/2/2) and moderate C4d immunoreactivity in the endothelial cells of hepatic (centrilobular) vein (400X); C: acute rejection in patients undergoing liver transplant for reasons other than hepatitis C (RejHCV-) group case with severe acute rejection (Banff 3/3/3). Despite moderate background staining in hepatocytes, C4d immunoreactivity in the endothelial cells of hepatic (centrilobular) vein is quite evident (200X); D: chronic hepatitis C in patients in a non-transplant setting (HCVTx-) group case staged as representing Ishak grade 1 fibrosis and grade 2 periportal inflammation with C4d immunoreactivity in the endothelial cells of portal vein branches (400X); E: RejHCV- group case with severe acute rejection (Banff 2/2/3) presenting C4d immunoreactivity in endothelial cell membranes of pericentral sinusoids (400X); F: HCVTx- group case staged as representing Ishak grade 5 fibrosis and grade 0 periportal inflammation with C4d immunoreactivity in endothelial cell membranes of periseptal sinusoids (400X).

Table IV shows the quantification of C4d deposition in the groups. In multiple comparisons, the HCVTx- group presented the greatest deposition (p = 0.003 vs. the RejHCV+ group, p < 0.001 vs. the HCVTx+ group and p = 0.019 vs. the RejHCV- group).

We found that portal immunostaining for C4d correlated significantly with continuous and categorical variables: total ischemia time (negative correlation, *r* = -0.244, p = 0.043), portal fibrosis (positive correlation, *r* = 0.571, p < 0.001), portal inflammation (positive correlation, *r* = 0.356, p = 0.011) and periportal inflammation (positive correlation, *r* = 0.336, p = 0.017). In the logistic regression model for the multivariate analysis, portal C4d positivity was found to be independently associated with the HCVTx- group (p = 0.016) and with periportal inflammation (p < 0.001).

*Quantification of HCV RNA in tissue* - Tissue samples tested positive for HCV RNA in 6 (21.4%) of the 28 RejHCV+ group cases, in 18 (78.2%) of the 23 HCVTx+ group cases and in only one (4%) of the 25 HCVTx- group cases. In multiple comparisons, HCV RNA levels were higher in the HCVTx+ group than in the RejHCV+ group (p < 0.001) (Table IV).

**TABLE IV:** Comparison of C4d deposition quantification and hepatitis C virus (HCV) RNA quantification in tissue by group

Variable	Group	n	Mean (range)	p^*a*^
Portal C4d^*b*^	RejHCV+	28	11.96 (0-50)	0.002
	HCVTx+	25	6.00 (0-20)	
	RejHCV-	20	12.75 (0-50)	
	HCVTx-	25	21.20 (0-70)	
Centrilobular C4d	RejHCV+	28	3.93 (0-50)	0.052
	HCVTx+	25	0.00	
	RejHCV-	20	5.50 (0-60)	
	HCVTx-	25	0.40 (0-5)	
Sinusoidal C4d	RejHCV+	28	0.54 (0-5)	0.206
	HCVTx+	25	0.00	
	RejHCV-	20	0.50 (0-10)	
	HCVTx-	25	0.80 (0-5)	
Tissue HCV RNA (IU/mL)	RejHCV+	28	120.50 (25-1.300)	< 0.001
	HCVTx+	23	1610.46 (25-15.900)	
	RejHCV-	25	26.50 (25-62.4)	

a: Kruskal-Wallis test; b: no difference was found among groups acute rejection in patients undergoing liver transplant for hepatitis C (RejHCV+), hepatitis C recurrence in patients undergoing liver transplant for hepatitis C (HCVTx+) and acute rejection in patients undergoing liver transplant for reasons other than hepatitis C (RejHCV); HCVTx-: chronic hepatitis C in patients in a non-transplant setting.

Positivity for HCV RNA in tissue was found to correlate significantly with categorical and continuous variables: patient age (positive correlation, *r* = 0.297, p = 0.009), time from transplantation to biopsy (positive correlation, *r* = 0.423, p = 0.002) and portal fibrosis (negative correlation, *r* = -0.440, p = 0.002). In the multivariate analysis regression model, the independent factors for higher HCV RNA levels included the time from transplantation to biopsy and belonging to the HCVTx+ group (p < 0.001 for both).

The ROC curve for the tissue level of HCV RNA presented an area under the curve of 0.818 (95% confidence interval 0.695-0.942). Table V shows the sensitivity, specificity, positive predictive values and negative predictive values with a selected cut-off point of 58.15 IU/mL. According to the ROC curve data, in the presence of suggestive morphological lesions, the specificity of the quantitative PCR of HCV RNA for diagnosing hepatitis C recurrence was 100% for values higher than 1.410 IU/mL. We also found a statistically significant correlation between the tissue level and serum level of HCV RNA (*r* = 0.391, p = 0.039).

**TABLE V:** Accuracy of hepatitis C virus RNA quantification in tissue for diagnosing hepatitis C recurrence with a selected cut-off point of 58.15 IU/mL

Test characteristic	Performance	95% CI
Accuracy	0.80	-
Sensitivity	0.70	0.47-0.87
Specificity	0.89	0.72-0.98
Positive predictive value	0.84	0.60-0.97
Negative predictive value	0.78	0.60-0.91
Positive likelihood ratio	6.49	2.16-19.6
Negative likelihood ratio	0.34	0.18-0.64

CI: confidence interval.

## DISCUSSION

The differential diagnosis between acute rejection and hepatitis C recurrence is of great importance in the post-operative follow-up of liver transplant recipients (Burton Jr & Rosen 2006). In the present study, there was no significant difference between the biopsy samples collected from the acute rejection patients and those collected from the hepatitis C-recurrent patients, in terms of the quantity of C4d deposition. However, the quantification of HCV RNA in tissue showed good accuracy for the diagnosis of hepatitis C recurrence.

Our findings corroborate those of Fayek (2012), who found that C4d staining was not able to differentiate between acute rejection and hepatitis C recurrence. However, previous studies, including one conducted by Schmeding et al. (2006), have suggested that C4d staining plays a major role in differentiating between acute rejection and hepatitis C recurrence. In a subsequent study, Schmeding et al. (2010) used ELISA for C4d detection and did not identify any differences between the acute rejection and hepatitis C-recurrent groups in terms of the C4d levels. In both studies, the authors evaluated a small number of biopsies. However, other studies, which were also based on a small number of biopsies, have reported that C4d expression plays an important role in the differential diagnosis between acute rejection and hepatitis C recurrence (Jain et al. 2006, Lorho et al. 2006).

Differences between our patient groups, in terms of the demographic, clinical and laboratory data, do not appear to have affected our results. In the RejHCV+ and HCVTx+ groups, the recipient and donor ages were similar. Although the time from transplantation to biopsy was longer in the HCVTx+ group patients, those patients required a definitive diagnosis and histological diagnosis that was considered to be the gold standard diagnostic method. Consequently, a diagnosis of acute rejection was more likely in the patients in whom the biopsies were performed within the first two months post-transplant, whereas a diagnosis of hepatitis C recurrence was more likely in the patients in whom the biopsies were performed more than six months post-transplant. Although we excluded the samples in which there were signs of both rejection and hepatitis C recurrence, such cases could be included in subsequent studies for validation purposes.

Considerable C4d deposition was observed in the HCVTx- group. In a non-transplant patient study conducted by Soglio et al. (2008), the biopsies tested positive for C4d in 40% of the chronic hepatitis C cases, 89% of the chronic hepatitis B cases and 83% of the autoimmune hepatitis cases. The authors suggested that C4d is not a useful marker for discriminating between acute rejection and hepatitis C recurrence. Other authors have observed C4d positivity in patients with chronic hepatitis B, autoimmune hepatitis or steatohepatitis, which calls into question the reliability of C4d as a marker of humoral rejection (Bu et al. 2006, Rensen et al. 2009, Aguilera et al. 2011).

During the pathogenesis of liver fibrosis, the innate and adaptive immune responses both play important roles (Hernandez-Gea & Friedman 2011) and the complement system is known to be involved in the pathogenesis of chronic hepatitis C (Dunkelberger & Song 2010). In a study assessing the mechanisms of cold activation of the complement system, Ishii et al. (2001) found that C4d deposition was greater in chronic hepatitis C patients than in HCV-negative patients, suggesting that the classical and lectin pathways are both activated in the pathogenesis of hepatitis C. 

Although previous studies have demonstrated the involvement of the complement system in HCV-induced liver disease, such studies have detected the presence of other products of the complement pathway (Pham et al. 1995, Hillebrandt et al. 2005, Brown et al. 2010, Banerjee et al. 2011), such as the membrane attack complex, as well as interactions between the E1 and E2 HCV glycoproteins and between C5 and the C5a receptor.

Given that the portal compartment demonstrated the greatest amount of C4d deposition, we performed a univariate analysis to identify the factors associated with C4d positivity in this compartment. The results indicated that portal immunostaining for C4d was associated with portal fibrosis, portal inflammation and periportal inflammation. However, after observing that the advanced stages of fibrosis and inflammation were more common in the HCVTx- group, we performed multivariate analysis, which demonstrated that the only independent factors were periportal inflammation and belonging to the HCVTx- group. This result highlights a limitation of our study, namely that fibrosis and portal and periportal inflammation grading differed between the hepatitis groups (HCVTx+ and HCVTx-).

In a recent study (published after the present study was conducted), Kozlowski et al. (2012) advocated immunofluorescence staining of frozen sections as the most reliable method for assessing C4d deposition in liver allograft biopsies. In cases of kidney transplantation, immunofluorescence detection using monoclonal antibodies in frozen tissue demonstrated better detection performance than did the use of polyclonal antibodies and immunohistochemistry in paraffin-embedded tissue, with a loss of C4d positivity (from diffuse to focal and from focal to minimal or negative) in 30% of the cases (Seemayer et al. 2007). Additionally, a recent multicentre study employing the Banff C4d schema (Mengel et al. 2013 ) showed poor inter-institutional reproducibility of C4d staining with immunohistochemistry in paraffinised sections obtained from renal allograft biopsies, which was attributed to limitations in technique and a lack of inter-rater concordance.

There is also significant heterogeneity among studies regarding the descriptions of grading and the site of C4d deposition (Krukemeyer et al. 2004, Dankof et al. 2005, Sawada et al. 2005, Bu et al. 2006, Jain et al. 2006, Lorho et al. 2006, Schmeding et al. 2006, Sakashita et al. 2007, Aguilera et al. 2011, Kozlowski et al. 2011, Musat et al. 2011, Lunz et al. 2012). In general, it has been suggested that positivity should be defined only on the basis of the diffuse form of staining, which is commonly used to represent positivity in more than 50% of the compartments. In the majority of previous studies, grading has been performed semi-quantitatively. As there is no consensus on the recommendations for this marker in liver transplantation, we aimed to obtain a more precise result by selecting an estimated quantification of C4d expression in each compartment. Using quantitative grading, we also considered focal positivity and the statistical analysis was performed according to quantitative results.

In the present study, we found no difference between specimens showing acute rejection and those showing recurrence of hepatitis C, in terms of C4d deposition, supporting the hypothesis that humoral mechanisms are involved in a small proportion of acute rejection episodes. However, those mechanisms also play a role in chronic hepatitis C, which makes it difficult to discriminate between these two conditions using C4d as a tissue marker. The subject of humoral rejection in liver transplantation has been the object of many recent studies, as well as the subject of Banff Conferences since 2011, as no specific consensus criteria exist for this entity in this population (Mengel et al. 2012a, b). Most recent studies have evaluated C4d positivity and its correlation with a positive HLA crossmatch by detecting donor-specific antibodies (Aguilera et al. 2011, Bellamy 2011, Kozlowski et al. 2011, Musat et al. 2011, Lunz et al. 2012). In fact, this mechanism highlights another limitation of the current study, which is that we did not perform concomitant detection of donor-specific antibodies, which would have been informative, especially in cases of C4d positivity. It has been suggested that the characteristic histological features of antibody-mediated rejection diffuse C4d positivity (present in > 50% of portal tracts or sinusoids) and the presence of donor-specific antibodies (Hübscher 2012).

In the context of the available literature, our data indicate the need for prospective, controlled clinical follow-up studies further assessing the role of C4d expression in each hepatic compartment, in formalin-fixed and frozen samples. Such studies could lead to the development of a more comprehensive assessment of pre and post-transplant crossmatching, using C4d positivity to assess each histological abnormality. Since the 2011 Banff Conference (Mengel et al. 2012b), experts have been discussing this possibility.

We found that HCV RNA levels were higher in the HCVTx+ samples than in the RejHCV+ samples, thus demonstrating good accuracy in predicting hepatitis C recurrence. These results corroborate those of previous studies, despite differences in the PCR techniques used (Aardema et al. 1999, Gottschlich et al. 2001, D'Errico-Grigioni et al. 2008). Because the time from transplantation to biopsy differed between the HCVTx+ and RejHCV+ groups, subsequent studies involving the quantification of HCV RNA should use paired samples in order to validate the HCV RNA level as a discriminator of the two diagnoses. In addition, the complexity of the technique must be considered before its use in clinical practice can be defined.

The fact that we observed HCV RNA positivity in 21.4% of the RejHCV+ group cases implies that tissue re-infection precedes morphological lesions in cases of recurrence, as suggested by Guerrero et al. (1997) (Hübscher 2012). In the HCVTx- group, there was a high rate of undetectable HCV RNA, which might have been attributable to prolonged storage of the samples in formalin, because this group was evaluated in a non-transplant setting where the formalin fixation time varies from 8-24 h, compared with 2 h in an urgent transplant setting. Additionally, previous studies have demonstrated lower rates of RNA detection, depending on the formalin fixation time (Guerrero et al. 1997). At the FMUSP Clinics Hospital, transplantation biopsy samples are processed within 2 h, whereas other biopsy specimens are formalin-fixed for 8-24 h. Using quantitative PCR, we found a correlation between serum and tissue levels of HCV RNA, which is consistent with the findings of previous studies in transplant and non-transplant settings (Martín et al. 1998, Nuovo et al. 2002, Descamps et al. 2012).

In conclusion, the role of C4d positivity in liver transplantation and HCV-related hepatic disease has yet to be fully explained. However, the current study demonstrates that HCV RNA quantification in tissue is an accurate method of diagnosing hepatitis C recurrence.
